# The impact of leukodystrophies on parents’ lives

**DOI:** 10.1093/jpepsy/jsaf072

**Published:** 2025-08-25

**Authors:** Laura Zampini, Laura Cordolcini, Lara Draghi, Paola Zanchi, Ylenia Vaia, Eleonora Bonaventura, Davide Tonduti

**Affiliations:** Department of Psychology, University of Milano-Bicocca, Milan, Italy; Department of Psychology, University of Milano-Bicocca, Milan, Italy; Department of Statistics, Computer Science, Applications (DiSIA), University of Florence, Florence, Italy; Department of Psychology, University of Milano-Bicocca, Milan, Italy; Unit of Pediatric Neurology, C.O.A.L.A. (Center for Diagnosis and Treatment of Leukodystrophies), V. Buzzi Children’s Hospital, Milan, Italy; Department of Psychology, Catholic University of the Sacred Hearth, Milan, Italy; Unit of Pediatric Neurology, C.O.A.L.A. (Center for Diagnosis and Treatment of Leukodystrophies), V. Buzzi Children’s Hospital, Milan, Italy; Department of Biomedical and Clinical Sciences, University of Milan, Milan, Italy; Unit of Pediatric Neurology, C.O.A.L.A. (Center for Diagnosis and Treatment of Leukodystrophies), V. Buzzi Children’s Hospital, Milan, Italy; Department of Biomedical and Clinical Sciences, University of Milan, Milan, Italy; Unit of Pediatric Neurology, C.O.A.L.A. (Center for Diagnosis and Treatment of Leukodystrophies), V. Buzzi Children’s Hospital, Milan, Italy; Department of Biomedical and Clinical Sciences, University of Milan, Milan, Italy

**Keywords:** leukodystrophy, caregiver burden, parenting stress, perceived social support, regression

## Abstract

**Objective:**

Leukodystrophies are a group of genetically determined neurological disorders affecting the white matter of the central nervous system and they have a profound impact on the daily lives of patients and their caregivers. However, only a few studies have analyzed the psychological experiences of parents of children with these conditions. The main aims of the present study were to assess parental burden and parenting stress in caregivers and to evaluate the relationships between parents’ experiences and the perceived clinical characteristics of their children.

**Methods:**

Forty-one parents of children and young adults diagnosed with leukodystrophies completed an online survey specifically designed to assess: the characteristics of parents and their children (i.e., current abilities, perceived severity level, and possible regression) and the psychological experiences of parents (i.e., caregiver burden, parenting stress, and perceived social support).

**Results:**

A significant proportion of parents who participated in the study were at risk of experiencing caregiver burden (63%) or parenting stress (49%). Regression analysis showed that perceived social support and the degree of regression (i.e., loss of competence) manifested by the children and young adults emerged as significant factors in determining caregiver burden. However, neither the severity of the child’s condition nor the age of the child/young adult appeared to be a determining factor in predicting parental burden or parenting stress.

**Conclusions:**

This study highlights the importance of considering parental well-being in both research and clinical practice, particularly for parents of children with progressive conditions.

Leukodystrophies are an increasingly recognized category of neurological disorders in pediatric neurology practice. They constitute a group of genetically determined disorders, affecting the white matter of the central nervous system with or without peripheral nervous system involvement’ (Sarret, 2020; Vanderver et al., 2015). Despite a wide range of clinical manifestations and diverse pathological mechanisms (Vanderver et al., 2015), they share a common impairment in the development, maintenance, or normal functioning of brain white matter (Bonkowsky et al., 2021). Determining the exact incidence of these diseases is challenging due to several factors (Ashrafi & Tavasoli, 2017; Vanderver et al., 2015), and the estimated incidence varies between studies, ranging from 1.2 in 100,000 (Vanderver et al., 2012) to 1 in 4,700 (Bonkowsky et al., 2021) live births. Moreover, recent advances in genetic testing suggest that leukodystrophies may be more prevalent than previously thought (Modesti et al., 2022).

Leukodystrophies can manifest at any stage of life, from the prenatal period to adulthood, with considerable variability in disease progression and clinical symptoms (Bonkowsky et al., 2021). In general, patients with leukodystrophy are profoundly affected by their condition and require assistance with basic activities of daily living. Initial symptoms are often nonspecific and affect the atypical acquisition of cognitive and motor milestones, leading to developmental delays in the early years of life (Modesti et al., 2022). In children and adolescents, it may present with behavioral, cognitive, or psychiatric changes, as well as the loss or deterioration of previously acquired milestones and skills (Bonkowsky et al., 2021). Diagnosis is challenging (Ashrafi & Tavasoli, 2017), and curative treatments for leukodystrophies are limited (e.g., hematopoietic stem cell transplantation or gene therapy) and not applicable to all types of leukodystrophies (Bonkowsky et al., 2021).

The prognosis of leukodystrophies cannot be generalized and must be determined for each specific type: While some leukodystrophies have a predictable course, others have a highly variable and unpredictable course. It has recently been reported that leukodystrophies show a mortality rate of more than 30% by the age of eight (Bonkowsky et al., 2021). Typically, an earlier age of onset is associated with greater disease severity and a more rapid decline (van der Knaap et al., 2019).

Leukodystrophies have a profound impact on patients’ daily lives, often requiring assistance with basic activities of daily living, which in turn can significantly increase the burden and distress of their caregivers. Therefore, when providing care, it is essential to consider the living environment and family context of affected children within a family-centered intervention framework (Park et al., 2018). This type of intervention prioritizes the family as the central unit of care. It recognizes families as experts on their children’s needs and their own circumstances, shifting the focus from a traditional professional-driven model to a collaborative partnership between professionals and families.

To date, only a few studies have analyzed the psychological experience of parents of children diagnosed with different types of leukodystrophies, highlighting that these caregivers might be at high risk for negative psychological experiences (Dermer et al., 2020; Lentini et al., 2025; Sevin et al., 2022; Thomas et al., 2024). Previous studies have shown that the progressive and degenerative features of leukodystrophies have a managerial (Brown et al., 2017), psychological (Eichler et al., 2016; Sevin et al., 2022), and social (Sevin et al., 2022) impact on the families of affected children, leading to noticeable changes in daily life (Eichler et al., 2016). These circumstances increase parental responsibilities and make the care of affected children constant and constantly changing, putting these parents at risk of increased stress (Dermer et al., 2020), lower quality of life (Lentini et al., 2025), and increased caregiver burden (Sevin et al., 2022; Thomas et al., 2024). To date, no clear relationships have been found between the parents’ experience and the severity of the disease or the presence of any regression in their children.

Parents of children with leukodystrophies might also report the need to leave their jobs, often due to concerns about the child’s physical health, their ongoing care needs or the desire to spend as much time as possible with their child (Ammann-Schnell et al., 2021). Additionally, these parents may need to consider the well-being of any siblings who could be affected, either directly or indirectly, by their brother or sister’s illness (Ammann-Schnell et al., 2021). In particular, mothers appear to experience higher levels of stress than fathers, probably due to their more frequent role as the child’s primary caregiver (Dermer et al., 2020).

Overall, this condition can cause parents to perceive a mismatch between their available resources and their child’s needs or demands. This is one of the most influential explanations of parental distress in the literature (Abidin, 1995) and it falls within the theoretical framework of transactional model of stress and coping devised by Lazarus & Folkman (1984), which posits that stress is a result of the perceived imbalance between environmental demands and one’s resources to cope with those demands. This model emphasizes that stress arises not just from external events (e.g., the child’s clinical condition), but from the subjective evaluation of these events (e.g., the perceived severity of the child’s clinical condition) and one’s ability to cope.

Therefore, the aim of the present study is to analyze the psychological experience of parents of children with leukodystrophies in relation to their perception of the severity of their child’s condition and the perceived regression in the skills manifested by their child. In view of the above, the main objectives of the present paper are the following: (a) To detect clinical characteristics of infants, children, and adolescents with leukodystrophies, as perceived by their parents; (b) To measure caregivers’ distress, burden, and perceived social support; (c) To test the association between clinical characteristics of children with leukodystrophies and parents’ psychological experiences; (d) To assess the predictive role of clinical characteristics of children with leukodystrophies on caregivers’ burden and parenting stress.

We expect to find high levels of individual variability in both the perceived clinical condition of children and parental experience. In particular, we expect to find higher stress and burden levels in parents of children with more severe conditions, as perceived by their parents. We also expect severity, regression, and children’s age to have a significant positive impact on caregivers’ burden and parenting stress.

## Methods

### Participants and procedure

Participants were asked to fill in an online survey specifically designed and made available as a public link through the websites and newsletters of national associations dealing with leukodystrophies, or through emails sent to the families followed by the Centre for Diagnosis and Treatment of Leukodystrophies and Genetic Leukoencephalopathies (C.O.A.L.A.) of the V. Buzzi Children’s Hospital (Milan, Italy). The public link was active for 1 year, from May 2023 to May 2024.

The survey was created and distributed using Qualtrics (https://www.qualtrics.com). The questionnaire was anonymous and took about 30 min to complete. The study was approved by the local commission for minimal risk studies of the Department of Psychology of the University of Milan-Bicocca (RM-2022-502). All parents signed an informed consent form before being included in the study.

The only inclusion criterion was being the parent of a child or a young adult with a leukodystrophy. A total of 51 parents had access to the questionnaire. Six of these parents did not consent to participation or data treatment, and four parents were excluded from the analysis because they did not answer all the survey questions. Therefore, 41 parents of children and young adults diagnosed with leukodystrophy participated in the study. Only one parent per child participated in the study to ensure the cleanliness of the data. The three topics investigated by the survey are described in the following paragraphs.

#### Characteristics of parents of children and young adults with leukodystrophy

We collected some personal (age, sex) and sociodemographic data (level of education, occupation, number of children). We then asked the parents whether they lived with their child with leukodystrophy and with the other parent of the child. Finally, we asked them, on average, how many hours a day from Monday to Friday they spent caring for their child with leukodystrophy, asking them to choose between the following alternatives: (a) 0–4 hr; (b) 4–8 hr; (c) 8–12 hr; (d) 12–24 hr.

#### Characteristics of children and young adults with leukodystrophy

This topic was analyzed through the following areas:

1. Personal data (age, sex, education) and clinical history data concerning leukodystrophy (type of diagnosis, time passed since the diagnosis);
2. Current competencies. Parents were asked whether or not their child was able to: (a) control his head; (b) sit up unsupported; (c) ambulate independently; (d) manipulate objects; (e) respond if called upon; (f) use gestures to communicate; (g) use words to communicate. Each one of these skills was considered as a dichotomic variable (0=not able; 1=able). In addition, parents were asked to describe how their child communicates by selecting the nearest alternative from the following: (a) does not communicate; (b) communicates only with gaze; (c) can use gestures, images, or symbols; (d) can use only a few words; (e) expresses himself through single words; (f) can produce simple word combinations; (g) can produce simple sentences (e.g., subject+verb+complement); (h) can produce complex sentences.
3. Parent perceived severity of symptoms. To evaluate the severity of symptoms shown by children and young adults, as perceived by their parents, we used an adapted version of a scale developed by the Authors and previously employed in studies involving parents of children with various medical and genetic conditions (e.g., XYY syndrome (Zampini et al., 2024), osteogenesis imperfecta (Aratti & Zampini, 2024), and genetic syndromes (Zampini et al., 2025)). The participants were asked to indicate if their children showed some problems in 15 areas of development: (a) sight; (b) hearing; (c) autonomous breathing; (d) autonomous feeding; (e) sphincter control; (f) self-care (e.g., dressing oneself); (g) upper motor skills; (h) lower motor skills; (i) language and communication; (j) reasoning; (k) attention; (l) behavior control; (m) emotional control; (n) relationship with others; (o) learning (e.g., reading and writing). For each of these areas, the parents were asked to rate on a four-point Likert scale whether their children showed problems or not as follows: (a) no (0 points), (b) mild (1 point), (c) medium (2 points), (d) severe (3 points). Parents were asked to report “not applicable” if their children were too young for a certain skill (e.g., academic learning if they had not yet started school). A perceived severity score [Severity score], ranging from 0 to 45, was then calculated by summing the scores in each area (“not applicable” was counted as “0” when calculating the total score because it was not possible to identify this type of difficulty at the time of compilation). We opted for this scale rather than other standardized instruments because it enables us to develop a specific scale for regression as perceived by parents. The “Parent perceived severity of symptoms” scale showed an excellent internal consistency (Cronbach’s alpha=.92).
4. Parent-perceived level of regression. To evaluate the eventual regression experienced by children and young adults with leukodystrophy, parents were asked to rate whether their children had manifested the loss of certain skills they had previously acquired and, if so, to what degree. They were also asked to indicate “not applicable” (counted as “0”) if their children had not yet acquired this competence. The same areas and Likert scale used to calculate the Severity score were used. Therefore, the perceived level of regression [Regression score] was calculated by summing the scores in each area, with a range from 0 to 45. The “Parent perceived level of regression” scale showed an excellent internal consistency (Cronbach’s alpha=.94).

#### Parents’ psychological experience

This topic was analyzed through the following areas:

1. Caregiver burden, as assessed by the Caregiver Burden Inventory (CBI) (Marvardi et al., 2005; Novak & Guest, 1989). This questionnaire consists of 24 items, divided into five dimensions of burden: (a) time-dependent burden [CBI Time-dependent] evaluating burden caused by the restriction of one’s personal time (e.g., “I don’t have a minute’s break from my caregiving chores”); (b) developmental burden [CBI Developmental] assessing the sense of failure regarding one’s hopes and expectations (e.g., “I wish I could escape from this situation”); (c) physical burden [CBI Physical] evaluating physical stress and somatic symptoms (e.g., “I’m physically tired”); (d) social burden [CBI Social] assessing the conflict of roles concerning one’s job or family (e.g., “I’ve had problems with my marriage”); (e) emotional burden [CBI Emotional] evaluating possible embarrassment or shame caused by the children’s condition (e.g., “I feel angry about my reactions toward my care receiver”). Each subscale consists of five items, with a score system ranging from 0 (minimum burden) to 4 (maximum burden). Since the CBI Physical subscale consists of four items, a correction factor of 1.25 is applied to be compared with the other subscales. An index of total burden [CBI total], ranging from 0 to 100, can be computed by summing the scores in the five subscales. Scores between 0 and 24 indicated a low risk for burden, scores between 25 and 36 indicated a moderate risk and scores over 36 indicated a high risk for burden. The CBI showed an excellent internal consistency (Cronbach’s alpha=.90).
2. Parenting stress, as assessed by the Parenting Stress Index short form (PSI) (Abidin, 1995; Guarino et al., 2008), which consists of 36 items to measure parenting stress through 3 subscales: parental distress, difficult child, and dysfunctional parent–child interaction. In the present study, we considered the total stress index calculated by adding the scores of the three subscales [PSI total]. Each of the 36 items rates from 1 (strongly disagree) to 5 (strongly agree). Raw scores (ranging from 36 to 180) were converted into percentiles, with a clinical range above the 85th percentile. The PSI showed an excellent internal consistency (Cronbach’s alpha=.93).
3. Perceived social support, as assessed by the Multidimensional Scale of Perceived Social Support (MSPSS) (Busoni & Di Fabio, 2008; Zimet et al., 1988), which consists of 12 items to measure perceived environmental support through 3 subscales: support from friends, support from family, and support from a significant other. The questionnaire includes four items for each source of support on a 7-point rating scale ranging from 1 (very strongly disagree) to 7 (very strongly agree). In the present study, we considered the composite score of perceived social support obtained by averaging the three subscales [MSPSS total] and ranging from 1 to 7. Higher scores indicate higher levels of perceived social support. The MSPSS showed an excellent internal consistency (Cronbach’s alpha=.90).

### Data analysis

Data analyses were carried out using IBM SPSS version 29. First, descriptive analyses of the characteristics of the parents and their children were reported. Second, parents’ psychological experiences were described and compared with normative data or clinical cut-offs. Thirdly, Pearson’s correlation was used to examine the relationships between psychological experience and parent and child characteristics. Finally, two regression analyses were performed to assess the predictive role of the investigated variables (i.e., children’s age, perceived severity and regression, and perceived social support) on caregiver burden and parenting stress.

## Results

### Characteristics of parents of children and young adults with leukodystrophy

As reported in the participants section, 33 mothers (80.5%) and 8 fathers (19.5%) completed the survey. They were all Italian, with a mean age of 43 years (*SD* = 8.03; range = 29–60) and a mean number of children of 1.78 (*SD*=.76; range = 1–4). In terms of educational level, 7 parents (17%) had attended primary and junior school (8 years of education), 19 (46%) had completed high school (13 years of education), 15 (37%) had completed university (16–18 years of education). In terms of occupation, 26 participants (63%) were working at the time of the study, while the other 15 (37%) were not working (e.g., homemaker, familiar caregiver, or looking for work). All parents reported living full-time with their child with leukodystrophy, except for one father who reported living 50% of the time because he and the other parent shared custody. Thirty-six parents (88%) reported living with the other parent, while five (12%) did not.

Regarding the time spent caring for their child with leukodystrophy, 10 parents (24%) reported caring for their child on average between 0 and 4 hr per day on weekdays, 8 parents (20%) 4–8 hr, 6 parents (15%) 8–12 hr, and 17 parents (41%) 12–24 hr per day.

### Characteristics of children and young adults with leukodystrophy

The age of the children and young adults ranged from 6 months to 25 years (*M* = 10.26 years; *SD* = 7.06). Twenty-nine (71%) were male and 12 (29%) were female. Three children (7%) attended nursery school, 4 (10%) kindergarten, 14 (34%) primary school, 4 (10%) junior high school, 4 (10%) high school, and 12 (29%) did not attend school or any other educational institution.

**Figure 1.**
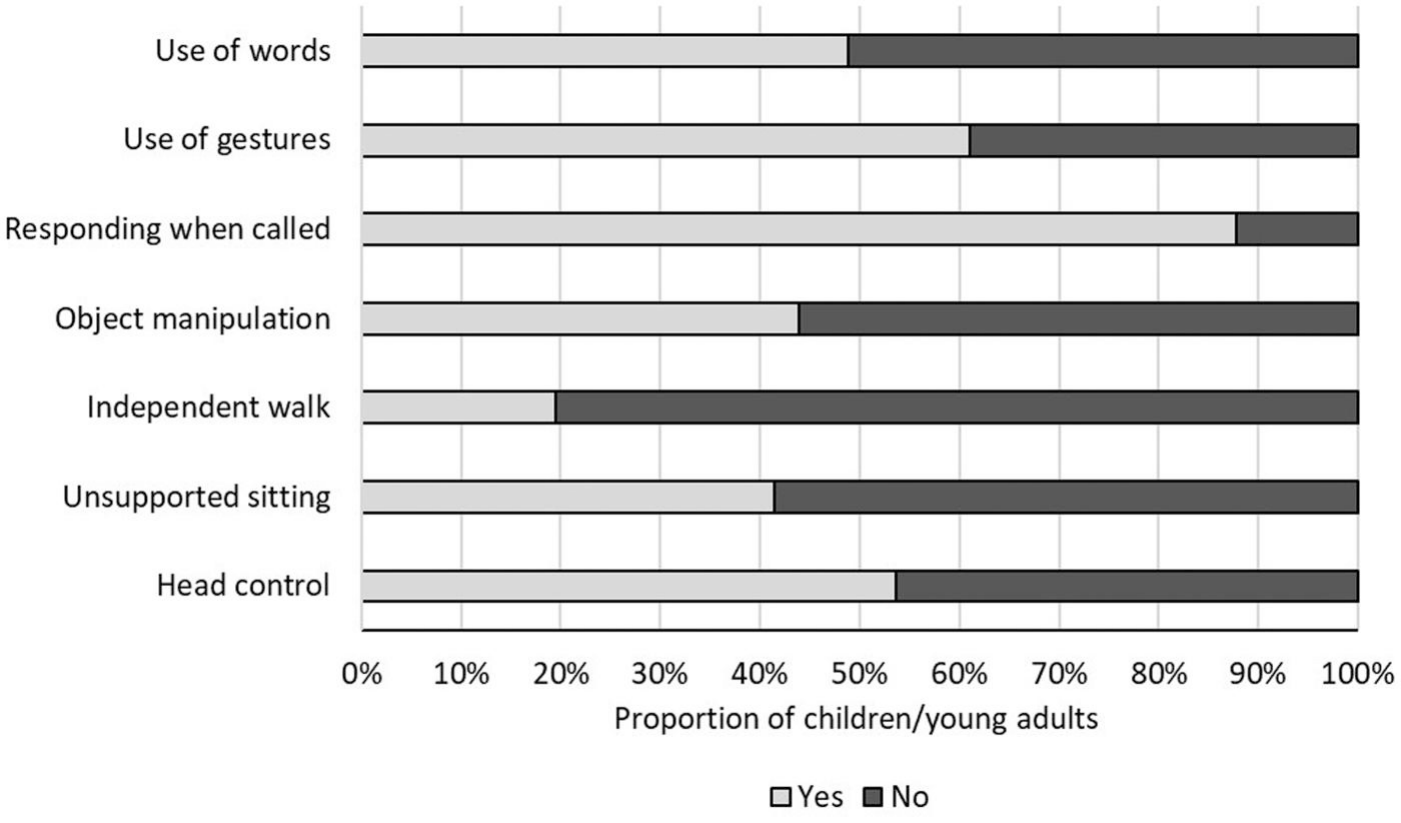
Proportion of children and young adults who show the listed skills at the time of the study.

**Figure 2.**
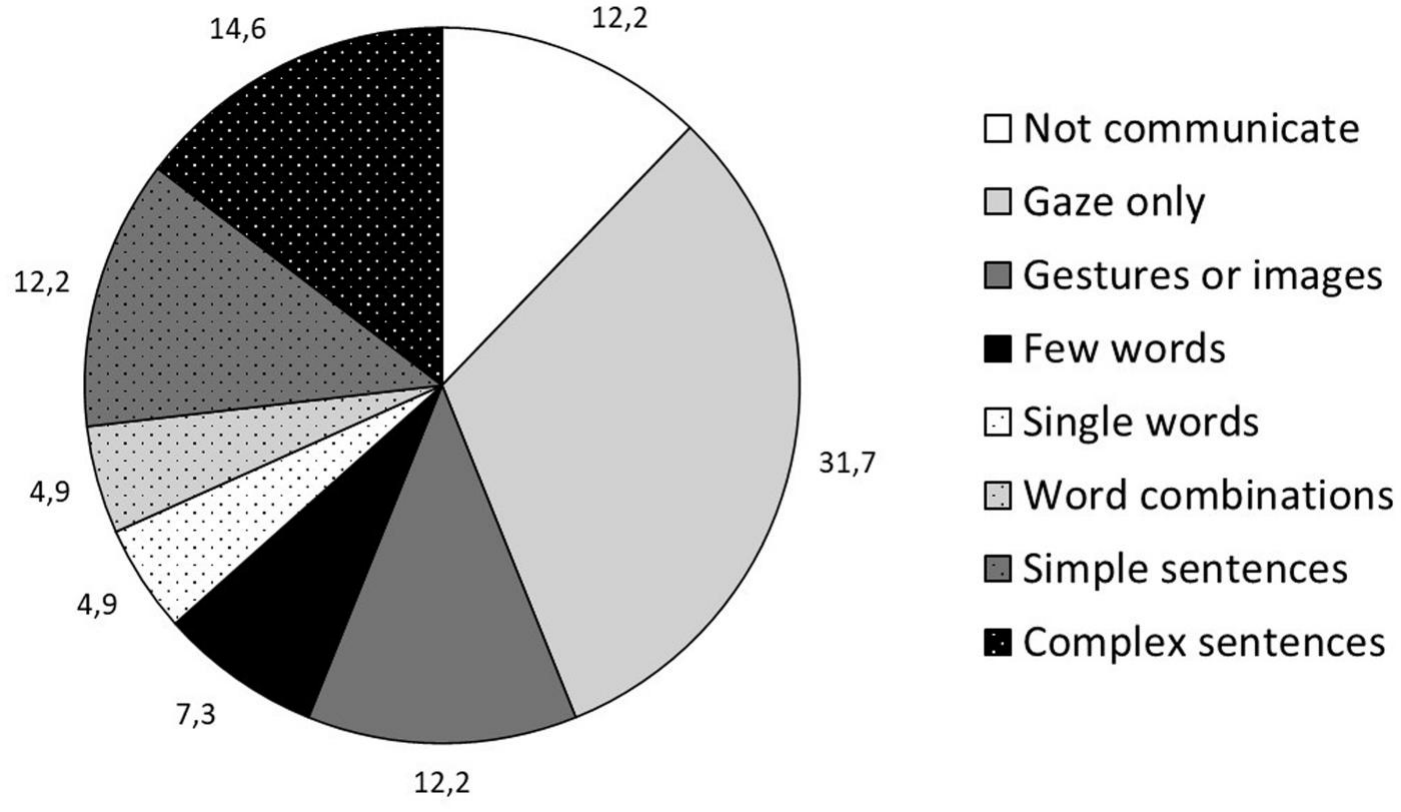
Proportion of children and young adults in each communicative skill level at the moment of the study.

The different types of leukodystrophies diagnosed and their relative frequencies are shown in Table 1. A mean of 84.51 months (i.e., almost 7 years) had elapsed since the parents were informed of the diagnosis (*SD* = 63.85; range = 1–240 months). With regard to the skills achieved, the motor and communicative skills at the time of the study are shown in Figures 1 and 2. Notably, less than 20% of participants could walk independently, and fewer than 50% could communicate using words. Furthermore, it should be noted that almost 44% of participants were reported as unable to communicate at all, or able to communicate only through gaze. Parents reported a mean Severity score of 17.32 (*SD* = 11.23; range 0–40), showing a large individual variability in the difficulties experienced by their children (the maximum score possible was 45). The areas with the most problems were self-care (*M* = 2.15; *SD* = 1.18; range = 0–3), academic learning (*M* = 2.10; *SD* = 1.21; range = 0–3), and lower limb motor skills (*M* = 2.03; *SD* = 1.24; range = 0–3).

**Table 1.** Types of leukodystrophies diagnosed in children and young adults.

Diagnosis	*N*	Proportion
Pelizaeus–Merzbacher disease	5	12.20
Alexander syndrome	4	9.76
X-linked adrenoleukodystrophy	3	7.32
Metachromatic leukodystrophy	3	7.32
L-2-hydroxyglutaric aciduria	2	4.88
Hypomyelination with atrophy of the basal ganglia and cerebellum (H-ABC)	2	4.88
POLR3-related leukodystrophy	2	4.88
Megalencephalic leukodystrophy with subcortical cysts type 1	2	4.88
Canavan disease	2	4.88
COL4A1/COL4A2 syndrome	2	4.88
Aicardi–Goutieres syndrome	2	4.88
TMEM63A-related leukodystrophy	1	2.44
Allan–Herndon–Dudley syndrome	1	2.44
Not specified ^a^	10	24.39

a Parents reported a diagnosis of leukodystrophy without specifying the type.

For the Regression score, a mean of 9.27 (*SD* = 10.94; range = 0–36) was reported; the variability in this score showed that some children and young adults did not show any regression, whereas others experienced a great loss of competencies acquired during their development (the maximum score possible was 45). The areas where the main regressions were found were language and communication (*M* = 1.21; *SD* = 1.34; range = 0–3), upper limb motor skills (*M* = 1.14; *SD* = 1.27; range = 0–3), and lower limb motor skills (*M* = 1.11; *SD* = 1.30; range = 0–3).

### Parents’ psychological experience

Descriptive statistics of the scores obtained by parents at the administration of the CBI, PSI, and MSPSS are shown in Table 2. A high variability was found in each dimension. Concerning caregiver burden, the highest scores were found in time dependence burden and physical burden. Looking at the individual scores of CBI total, 26 parents (63%) appeared to be at risk of caregiver burden, of whom 12 (29%) were at moderate risk and 14 (34%) at high risk. In addition, 20 parents (49%) scored above the clinical range of the 85th percentile for parenting stress. No normative scores or cut-off points are available for the MSPSS, but the wide range of scores (3–7) indicates a high degree of variability in parental perception of social support.

**Table 2.** Descriptive statistics of caregiver burden, parenting stress, and perceived social support.

	*M*	SD	Range	*N* (%) of parents above cut-off scores
CBI total	30.22	14.40	0–66	26 (63%)
CBI Time-dependent	13.83	5.12	0–20	
CBI Developmental	5.88	4.70	0–18	
CBI Physical	6.98	5.98	0–20	
CBI Social	3.80	3.80	0–13	
CBI Emotional	1.12	1.58	0–6	
PSI total	65.29	31.60	1–100	20 (49%)
MSPSS total	5.07	1.13	3–7	

*Note*. CBI=Caregiver Burden Inventory (CBI total: *x* > 25 = moderate risk; *x* > 36 high risk); PSI=Parenting Stress Index (PSI total: *x* > 85 = clinical range); MSPSS=Multidimensional Scale of Perceived Social Support.

The three variables (i.e., CBI total, PSI total, and MSPSS total) appeared to be significantly related, as caregiver burden and parenting stress were positively correlated (*r*=.51; *p*<.001; *r*^2^=.26) and both negatively correlated with perceived social support (CBI-MSPSS: *r* = −.47; *p*=.002; *r*^2^=.22; PSI-MSPSS: *r* = −.32; *p*=.044; *r*^2^=.10), confirming the protective factor of social support. No associations were found between caregiver burden and parenting stress and parental age (CBI-parent age: *r*=.18; *p*=.261; *r*^2^=.03. PSI-parent age: *r* = −.08; *p*=.613; *r*^2^=.01), while children’s age was found to be significantly positively correlated with burden (*r*=.37; *p*=.016; *r*^2^=.14) but not with parenting stress (*r*=.16; *p*=.311; *r*^2^=.03). The child’s severity score was also found to be correlated with caregiver burden (*r*=.58; *p*<.001; *r*^2^=.34) but not with stress (*r*=.18; *p*=.264; *r*^2^=.03), while the regression score was found to be significantly correlated with both burden (*r*=.59; *p*<.001; *r*^2^=.35) and parenting stress (*r*=.32; *p*=.044; *r*^2^=.10).

Two regression analyses were then conducted to assess the factors that may explain parental burden and parenting stress. Independent variables included children’s age, perceived severity and regression, and perceived social support. All independent variables had Variance Inflation Factor (VIF) values <10 (range=1.16–2.37); therefore, multicollinearity was tolerable. The results, reported in Table 3, showed a significant role for the regression score and social support in explaining 43% of the variance in caregiver burden. Conversely, only 8% of the variability in parenting stress was explained, and the only predictor was the regression score (see Table 4). The age and severity of the children and young adults did not contribute to explaining variability in caregiver burden or stress.

**Table 3.** Predictive model of caregiver burden.

Model	Dependent variable	Entered variables	*R*²	Adjusted *R*^2^	*F* (df)	*ΔR*²	*β*
1	CBI total	Regression score	0.35	0.33	20.53 (1, 39)***	0.35	0.59***
2	CBI total	Regression score MSPSS total	0.46	0.43	7.72 (2, 38)***	0.11*	0.50*** −0.34**

*Note*. CBI=Caregiver Burden Inventory; Regression score=parent perceived level of regression; MSPSS=Multidimensional Scale of Perceived Social Support.

*  *p*>.05;

**  *p*>.01;

***  *p*>.001.

**Table 4.** Predictive model of parenting stress.

Model	Dependent variable	Entered variables	*R*²	Adjusted *R^2^*	*F* (df)	*ΔR*²	*β*
1	PSI total	Regression score	0.10	0.08	4.34 (1, 39)*	0.10	0.32*

*Note*. PSI=Parenting Stress Index; Regression score=parent perceived level of regression.

*  *p*>.05;

**  *p*>.01;

***  *p*>.001.

## Discussion

The main aim of the study was to assess the relationship between the perceived severity of one’s own child with leukodystrophy and the psychological experiences of parents. The first important fact to note is that about 40% of the participants indicated that they care for their child intensively on a daily basis (more than 12 hr per day). These data correspond to the percentage of parents who declared that they were not working at the time of the survey, probably because of the need to look after their children with leukodystrophy.

Wide individual variability was found in the level of severity perceived by parents and also in the level of regression of previously acquired skills manifested by children and young adults. This can be explained by the fact that the participants’ children have different types of leukodystrophy, are of different chronological ages, and are at different stages of the disease (some have been diagnosed recently, others for many years). For those who have experienced a regression, the loss of skills was most evident in the areas of language and communication and motor development. It is important to take this into account as the loss of the ability to speak has been identified as one of the most disturbing and worrying symptoms for caregivers of patients with metachromatic leukodystrophy (Eichler et al., 2016).

In this study, the use of standardized quantitative instruments revealed that a significant percentage of parents are at risk of experiencing caregiver burden (63% of the participants) or parenting stress (49% of the participants). This result confirms previous findings from qualitative research or studies on specific types of leukodystrophy (e.g., POLR3-related leukodystrophy or metachromatic leukodystrophy) that underlined how these parents show a low quality of life and a higher risk of stress (Dermer et al., 2020; Eichler et al., 2016; Lentini et al., 2025; Sevin et al., 2022).

Perceived social support appeared to be a protective factor, as found in parents of children with different diseases or conditions (Aratti & Zampini, 2024; Halstead et al., 2017; Pinquart, 2018). Specifically, the negative relationship between perceived social support and caregiver burden was stronger than the relationship between perceived social support and parenting stress.

Although children’s age and severity were positively correlated with caregiver burden, when considering the factors that may explain the extent to which parents feel overwhelmed (CBI) or stressed (PSI), it is interesting to note that neither the severity of the child’s condition nor the age of the child/young adult appears to be a determining factor. However, there are conflicting data in the literature on the relationship between parenting stress and the severity of children’s chronic illness, with some studies supporting a positive association between stress and severity (Pinquart, 2018) and others finding that parenting stress was unrelated to duration and severity of illness (Cousino & Hazen, 2013). This aligns with the transactional model of stress and coping proposed by Lazarus and Folkman (1984), which posits that stress is not merely a direct response to an objective stressor, but rather a dynamic process mediated by an individual’s cognitive appraisals. According to this model, parents would first engage in primary appraisal, evaluating the perceived severity of their child’s condition and the implications of skill regression as a potential threat or harm. Subsequently, secondary appraisal involves assessing their available coping resources, such as perceived social support, to manage the demands of caregiving. The variability in parental psychological experiences, despite similar objective circumstances, underscores the critical role of these subjective appraisal processes and individual coping strategies in determining the level of stress and burden experienced.

Both the perceived social support, as mentioned above, and the degree of regression (i.e., the loss of competence) manifested by the children and young adults emerged as significant factors in determining caregiver burden. Conversely, only the presence of a regression appeared to be a significant factor in determining the stress experienced by parents.

The findings of our study have important practical implications for families with children affected by leukodystrophies and for the wider healthcare policy framework. As perceived social support was found to be a significant protective factor against caregiver burden, it is crucial that families are informed about and connected with the relevant support networks proactively. These networks should include specialized leukodystrophy clinical centers offering expert counseling and adaptive care strategies, as well as clear information about the child’s specific diagnosis and prognosis. It is also important to facilitate access to family associations (such as the European Leukodystrophy Association—ELA or the United Leukodystrophy Foundation—ULF) and community groups to foster peer connections, as shared experiences can mitigate feelings of isolation.

Our data further emphasize the need for a more robust family-centered care approach for children with leukodystrophies. Within this framework, the psychological well-being of parents must be carefully evaluated and incorporated into the patient’s treatment plan as an essential component. Therefore, policy initiatives should prioritize the establishment of accessible, structured psychological support programs for caregivers. These programs should provide access to mental health professionals, facilitate participation in support groups, and offer respite care services to prevent burnout. Furthermore, strategies to actively cultivate and strengthen social support networks for these families must be systematically implemented. Given the significant time commitment involved in caregiving, with 40% of parents reporting over 12 hr per day, policy considerations must also encompass enhancing flexible work arrangements and developing comprehensive assistance programs. These measures are vital in alleviating the economic and logistical burden on families, enabling parents to devote the necessary time to their child’s care without facing undue hardship.

In conclusion, this study shows high levels of parenting stress and caregiver burden in the parents of patients with leukodystrophies. In particular, a high level of burden deriving from not having time for themselves (Time-dependent burden) has been found, probably because these parents have to devote a lot of their personal time to the special needs of their children. However, it should be emphasized that perceived social support might have a role in mitigating the effects of caregiver burden. Given that the well-being of the family has an important impact on the developmental outcome and well-being of children with neurological diseases (Etemadifar et al., 2018; King & Chiarello, 2014), it is essential that treatment planning for patients with leukodystrophies takes place within a context of family-centered care, in which direct care for the needs of the patient is indirectly supported by care for the needs of the family, particularly the parents who is daily involved in child care.

### Study limitations and future perspectives

The study has limitations. First, the small sample size makes it difficult to generalize the results to all parents of individuals with leukodystrophies. It should also be emphasized that leukodystrophies are a highly varied condition, with different levels of severity and times of diagnosis ranging from the prenatal period to adulthood. This makes generalization of results even more difficult. Secondly, the parents who responded to the questionnaire were in very different circumstances. Some were primary caregivers, while others were not, as evidenced by the number of hours they spent caring for their child each day. Some of these parents also stated that they do not live with the other parent; however, the questionnaire did not ask whether they were single or had a partner who helped with the daily care of their child.

In addition, it should be noted that, although the percentage of variance explained by the included variables is good (43%) for caregiver burden, it is very low (8%) for parenting stress. This suggests that other variables that we did not consider could contribute to explaining the variability in this factor. For instance, a limitation of the present study is that we did not ask the parents whether they had another child with leukodystrophy or any other clinical conditions. Future studies will look at other indices, such as parental personality traits, coping strategies, or the presence of other protective factors (such as the presence of other healthy children), to investigate their impact on parental well-being.

Lastly, the fact that standardized tools were not used to assess parents’ perceptions of their children, in addition to the specially designed scales, could be seen as a limitation of this study. Future studies could correlate parents’ perceived severity of symptoms and perceived level of regression with the results of standardized questionnaires for assessing children’s adaptive functioning. Examples of such tools include the Vineland Adaptive Behavior Scales and the Adaptive Behavior Assessment System (ABAS).

Despite its limitations, this study highlights the importance of considering the well-being of parents and the need to develop support and guidance programs, particularly for parents of children with conditions characterized by progressive loss of abilities.

## Data Availability

None declared.
